# Elevated Serum Ferritin Is Associated with Reduced Survival in Amyotrophic Lateral Sclerosis

**DOI:** 10.1371/journal.pone.0045034

**Published:** 2012-09-14

**Authors:** Yann Nadjar, Paul Gordon, Philippe Corcia, Gilbert Bensimon, Laurence Pieroni, Vincent Meininger, François Salachas

**Affiliations:** 1 Fédération des Maladies du Système Nerveux, APHP, Centre de référence maladies rares SLA, Hôpital Pitié-salpêtrière, Paris, France; 2 Centre SLA, CHU de Tours, Université François Rabelais, Tours, France; 3 Département de Pharmacologie Clinique, Hôpital Pitié-salpêtrière, et UPMC Pharmacologie, Paris 6, UMR 7211, Paris, France; 4 Biochimie Métabolique, APHP, Hôpital Pitié-Salpêtrière, Paris, France; University of New South Wales, Australia

## Abstract

**Background:**

Amyotrophic lateral sclerosis (ALS) is a neurodegenerative disorder caused by the loss of motor neurons. Its etiology remains unknown, but several hypothesis have been raised to explain motor neuron death, including oxidative stress. Dysregulation of cellular iron metabolism can lead to increased oxidative stress, and existing data argue for a role of iron metabolism in ALS pathophysiology.

**Methods:**

We performed a retrospective analysis of iron metabolism (IM) variables (serum levels of iron, transferrin, ferritin, and TSC for Transferrin Saturation Coefficient) in a cohort of 694 ALS patients and 297 healthy controls.

**Results:**

Serum ferritin levels and TSC were higher, whereas serum transferrin levels were lower in ALS patients than controls. In addition, patients with a high level serum ferritin had a shorter survival time compared to those with low level serum ferritin (618 days versus 921 days for men subgroup; p = .007). Site of onset and ALS-FRS score were not associated with IM variables.

**Conclusion:**

This study suggests that ALS patients may have increased iron storage, as measured by increased serum ferritin and TSC. Elevated serum ferritin may also have a deleterious impact on survival in ALS.

## Introduction

Amyotrophic Lateral Sclerosis (ALS) is a neurodegenerative disorder caused by the loss of motor neurons, leading to progressive weakness. Death generally occurs within 2–5 years after symptoms onset, most often due to respiratory failure. The only drug approved for treatment, riluzole, has a modest effect on survival. [1] Ten percent of cases are familial with mutations in the gene encoding Superoxide dismutase 1 (SOD1) accounting for 20% of familial ALS. Familial and sporadic ALS usually have similar phenotype.

The etiologies of sporadic ALS are unknown, but pathological studies in humans and the murine model of ALS (SOD1 mutated mouse) have exposed several physiopathological pathways, including oxidative stress. [2].

Defects in the cellular regulation of iron metabolism can lead to increased intra cellular oxidative stress through the production of reactive oxygen species (ROS), and iron metabolism may be abnormal in ALS. [3] Increased concentrations of iron are found in the cell bodies of motor neurons. [4] Transferrin, a protein involved in iron transport, is found in ALS specific Bunina’s bodies located in motor neurons. [5] A polymorphism of the HFE gene, responsible for genetic hemochromatosis, may be a risk factor for ALS. [6]–[9] Serum ferritin, a marker of iron storage, may also be increased in ALS patients.[10]–[12] In the murine model, iron accumulates in motor neurons, where iron related proteins are upregulated, and iron chelator treatment leads to improved survival that correlates with decrease in iron accumulation. [13].

In this retrospective study we analyzed the iron metabolism (IM) variables serum iron, ferritin, and transferrin as well as the transferrin saturation coefficient (TSC) in a cohort of ALS patients and controls.

## Patients and Methods

We analyzed data from 694 consecutive patients with definite, probable or laboratory probable ALS at the time of blood sampling who were evaluated at the national referral centre for ALS in Paris between 1994 and 1999. [14].

All patients underwent systematic laboratory testing, including serum iron, serum transferrin, and serum ferritin, performed as part of the new patient evaluation. Signed consent was not requested for the tests because they were a part of clinical care, as approved by the ethical committee of Pitié Salpétrière Hospital (CPPRB, for Comité de Protection des Personnes participant à la Recherche Biomédicale).

**Table 1:** General characteristics of healthy controls and ALS populations.

	Healthy controls (n = 297)	ALS (n = 694)		
		serum iron (n = 694)	serum transferrin (n = 677)	serum ferritin (n = 629)
sex ratio (male/female)	1.4/1	1.08/1	1.08/1	1.05/1
Mean age at draw, years (SD)	48.99 (13.01)	61.85 (12.00)	61.88 (12.06)	61.53 (12.14)
*p value for sex ratio (Controls vs ALS)*		*0.071*	*0.081*	0.048*
*p value for age (Controls vs ALS)*		<0.001*	<0.001*	<0.001*
Bulbar vs limb onset ratio		1/1.94	1/1.94	1/1.93

Three slightly different ALS populations are shown here. Among 694 ALS patients with a serum iron value available, serum transferrin values and serum ferritin values were available for 677 and 629 patients, respectively.

**Table 2:** IM variables levels according to gender and disease status (means with standard deviations).

	Men			Women		
	Controls (n = 173)	ALS (n = 360)	*p value*	Controls (n = 124)	ALS (n = 334)	*p value*
serum iron (micromol/L)	19.41 (5.18)	20.07 (6.59)	0.25	17.97 (6.67)	18.18 (5.7)	*0.74*
serum transferrin (g/L)	2.36 (0.32)	2.24 (0.41)	0.001*	2.52 (0.47)	2.37 (0.47)	0.07*
transferrin saturation coefficient (TSC) (%)	33.56 (10.13)	36.22 (13.91)	0.026*	29.43 (11.8)	31.78 (12.8)	0.002*
serum ferritin (microg/L)	180.46 (78.6)	228.56 (147.7)	<0.001*			

At time of blood sampling we recorded demographic data including age, sex, site of onset, date of onset of weakness, and ALSFRS (Amyotrophic Lateral Sclerosis Functional Rating Scale) score. Date of death was assessed by death certificate or written letter from relatives or physicians. The database was closed on April 1, 2009. On this date, 659 patients had died, 14 were alive, and 21 were lost to follow up. Given the high rate of mortality and low rate of lost to follow up, we included only deceased patients for survival analysis. Survival times were calculated in days from the time of blood sample to the date of death.

For comparison, we also analyzed data from 297 healthy controls who were recruited from the iron national referral centre at Hopital Pontchaillou in Rennes, where laboratory tests were performed.

**Table 3:** Serum ferritin levels for women according to age (< or > to 45 years old) and disease status (means with standard deviations).

	Women					
	< 45			> 45		
	Controls (n = 45)	ALS (n = 33)	*p value*	Controls (n = 77)	ALS (n = 274)	*p value*
serum ferritin (microg/L)	47.97 (45.52)	63.08 (68.7)	0.247	85.54 (61.86)	123.51 (104.59)	0.003*

For ALS patients, transferrin and iron levels were determined by immunoturbidimetry and by colorimetry using the ferrozin method, respectively, on an Integra 400 (Roche Diagnostics, Meylan, France) ; ferritin was determined by immunonephelometry on a nephelometer BN2 (Siemens, Germany), and transferrin saturation coefficient (TSC) was calculated according to the formula : iron(µmol/L)*100 / transferrin (g/L)*0.25. For healthy controls, the same technique was used to measure iron and transferrin, but ferritin was determined by chemiluminescence on a Centaur (Bayer diagnostics). Ferritin levels for twenty different sera were obtained by both methods (immunonephelometry in Paris and chemiluminescence in Rennes). The correlation coefficient for both methods was 0.99, allowing comparison of ferritin levels in ALS patients and healthy controls after applying a corrective factor of 0.83 for ALS patients’ ferritin values.

**Table 4:** IM variables levels in ALS patients according to site of onset (means with standard deviations).

	Bulbar (n = 236)	Lower limbs (n = 238)	Upper limbs (n = 220)	*p value*
serum iron (micromol/L)	18.33 (6.11)	19.61 (5.97)	19.57 (6.63)	*0.219*
serum transferrin (g/L)	2.29 (0.47)	2.28 (0.46)	2.32 (0.32)	*0.37*
transferrin saturation coefficient (TSC) %	32.5 (13.01)	35.23 (13.46)	34.57 (14.16)	*0.339*
serum ferritin (microg/L)	171 (138)	170 (132)	180 (139)	*0.417*

p values are adjusted for sex for serum iron, serum transferrin and TSC, and for sex and age for serum ferritin.

### Statistical Analysis

All continuous variables showed a normal distribution. Correlation was performed using Spearman’s correlation analysis to compare the two methods for serum ferritin measurement. Chi2 tests compared sex ratio between ALS and control groups. T-tests compared mean age and means of iron metabolism (IM) variables between ALS patients and controls. A generalized linear model compared IM variable levels by site of onset and controlling for gender and age when necessary. For each model, one IM variable was used as a continuous variable, while site of onset and gender were entered as categorical covariates. For analysis of serum ferritin only, age was also included as a covariate in the model. One way ANOVA compared mean ALSFRS score and survival time by tertiles for each IM variable. Kaplan Meier analysis generated survival curves for high (n = 297, >156 microg/L) versus low level serum ferritin (n = 297, <156 microg/L) in ALS patients. A Cox model was built to compare low and high level serum ferritin ALS populations with age and gender adjustment.

Differences were considered significant at p<0.05 (two-sided). Data were expressed as mean and standard deviation. Analyses were performed with the SPSS version 11.0 software.

**Figure 1:**
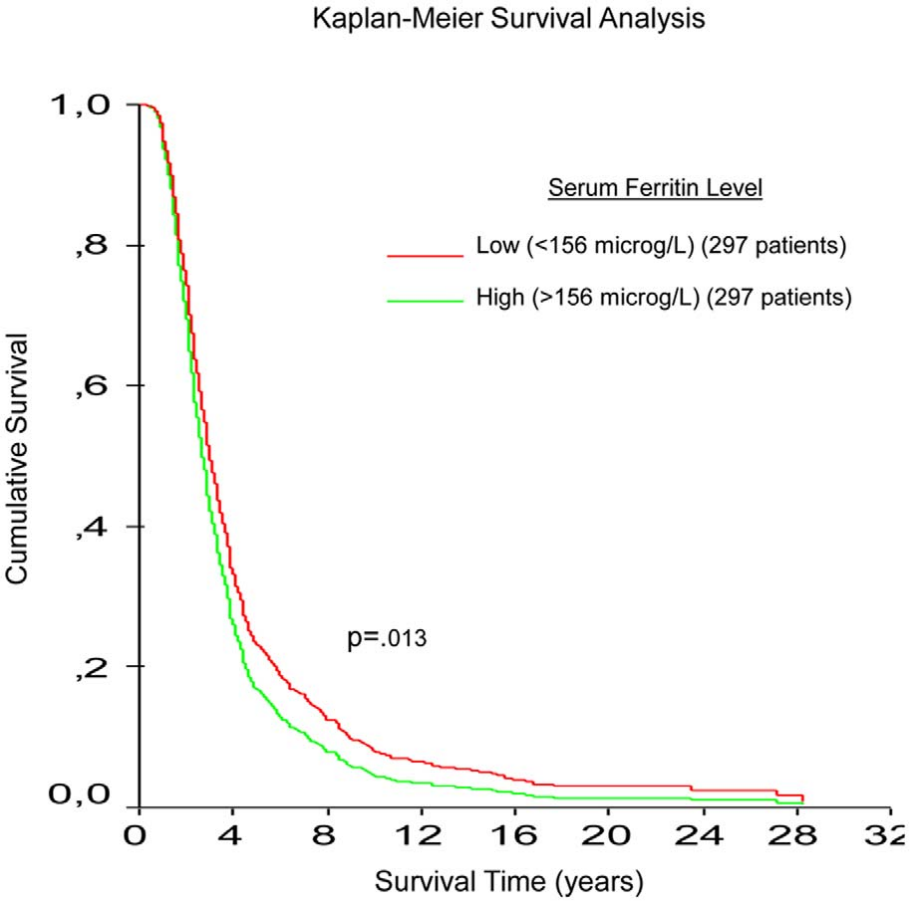
Kaplan Meier survival curves of ALS patients with low versus high serum ferritin level. Red curve includes ALS patients with ferritin level <156 microg/L (297 patients). Green curve includes ALS patients with ferritin level >156 microg/L (297 patients). p value is age and gender adjusted (Cox model).

## Results

In the 694 ALS patients, the sex ratio (M/F) was 1.07/1, mean age was 61.85 ± 12.00 years and ratio of site of onset (Bulbar/Limb) was 1/1.9 (Table 1). Seventeen patients (2%) did not have serum transferrin levels and 65 (9%) did not have ferritin levels, so the following analyses are based on 677 and 629 patients respectively. These groups had the same general characteristics as the overall population of ALS patients (Table 1).

**Table 5:** ALS-FRS scores in ALS patients according to gender and IM variables tertiles (low, medium and high levels) (means with standard deviations).

	Mean ALS-FRS score according to IM variables tertiles							
	Men				Women			
	Low	Medium	High	*p value*	Low	Medium	High	*p value*
serum iron	30.22 (6.04)	30.98 (5.01)	31.76 (5.16)	*0.33*	28.8 (4.63)	30.61 (4.97)	31.15 (4.62)	*0.05*
mean age (years)	62.8	60.7	59.4		64.8	64.1	62.9	
serum transferrin	31.05 (4.88)	30.19 (6.12)	32.23 (5.12)	*0.19*	29.91 (4.62)	30.75 (4.62)	30.56 (5.17)	*0.63*
mean age (years)	62.17	61.17	59.9		65.7	63.8	62.2	
transferrin saturation coefficient	30.47 (5.96)	31.2 (5.15)	31.43 (5.20)	*0.65*	29.29 (4.96)	30.67 (4.25)	30.73 (4.91)	*0.3*
mean age (years)	62.3	60.7	60		64.5	64.5	62.9	
serum ferritin	32.25 (5.22)	29.96 (5.63)	30.78 (5.29)	*0.1*	29.43 (6.02)	31.04 (5.42)	30.37 (4.48)	*0.38*
mean age (years)	59.8	59.8	62.4		59	64.4	67.2	

For each tertile group, mean age is indicated below ALS-FRS mean value.

Comparisons between ALS patients and the control group by age and gender showed ALS patients were older (iron, transferrin, ferritin subgroups) and contained more men but the difference reached significance (p = 0.048) only in the ferritin subgroup (Table 1).

**Table 6:** Survival times in ALS patients according to gender and IM variables tertiles (low, medium and high levels) (means with standard deviations, in days).

	Mean survival time according to IM variables tertiles (days)							
	Men (n = 337)				Women (n = 322)			
	Low	Medium	High	*p value*	Low	Medium	High	*p value*
serum iron	756 (840)	675 (601)	766 (725)	*0.6*	746 (776)	848 (900)	854 (844)	*0.56*
mean age (years)	62.8	60.7	59.4		64.8	64.1	62.9	
serum transferrin	633 (598)	741 (770)	809 (751)	*0.17*	696 (738)	914 (929)	869 (858)	*0.13*
mean age (years)	62.17	61.17	59.9		65.7	63.8	62.2	
transferrin saturation coefficient	839 (893)	658 (625)	678 (542)	*0.11*	866 (926)	692 (643)	903 (919)	*0.16*
mean age (years)	62.3	60.7	60		64.5	64.5	62.9	
serum ferritin	921 (897)	686 (646)	618 (629)	0.01*	964 (985)	775 (752)	671 (715)	0.04*
mean age (years)	59.8	59.8	62.4		59	64.4	67.2	

For each tertile group, mean age is indicated below survival time mean value.

In men (controls and ALS), no IM variable was correlated with age, whereas levels of serum ferritin increased in women up to 45 years of age (Tables S1 and S2). All IM variables were related to gender. In women, serum iron, TSC and serum ferritin were lower than in men, whereas serum transferrin was higher (Table S3). To avoid bias related to age and gender when comparing variables between control and ALS patients, subsequent statistical analyses were conducted separately according to gender, and also according to age for serum ferritin in women only.

Tables 2 and 3 shows comparisons between ALS patients and controls for levels of serum iron, ferritin and transferin. ALS patients had lower serum transferrin levels (significant in men) and higher TSC levels than controls. Serum iron levels were similar between cases and controls in both men and women. Serum ferritin was higher in male ALS patients than controls. In women, serum ferritin levels were higher only in ALS patients above age 45, possibly due to the low number of patients in under age 45 group (Table 3).

For each IM variable, no difference was observed for site of onset, after adjusting for gender and age (Table 4).

To examine correlations between clinical data (ALSFRS scores and survival time) with IM variables, we split ALS patients into tertiles for each IM variables (low level, medium level, high level). Cut off values and patient numbers in tertiles for each IM variables are given in Table S4. No significant differences were observed between ALSFRS scores and any IM variable (Table 5). Analysis of survival time showed that male patients with low levels of ferritin lived about 300 days longer than patients with medium or high levels (921 days vs 686 and 618 days respectively, p = .01). In women, high level serum ferritin was also associated with a reduced survival time (Table 6), but this association was no longer significant after adjustment for age. Figure 1 shows the Kaplan Meier survival curve for high (n = 297, >156 microg/L) versus low level serum ferritin (n = 297, <156 microg/L) in ALS patients. As expected, survival was reduced for high level serum ferritin patients (p = .013 after age and gender adjustment).

## Discussion

This retrospective study of 694 ALS patients and 297 healthy controls showed that ALS patients, compared to controls, have an increased serum ferritin level, increased TSC, and a decreased serum transferrin level. In addition, high level serum ferritin is associated with a decreased survival in men.

Three previous studies of fewer patients showed that ALS patients had increased serum ferritin levels, and one showed that ALS patients had decreased serum transferrin level. [10]–[12] Ferritin, expressed in all cells, is a principally intra cellular protein that forms a cytosolic sphere for iron storage. Ferritin prevents cellular oxidative reactions due to free iron, and is considered to have an anti-oxidative cellular function. [15] The function of serum ferritin is less well understood, but is widely used as an indicator of body iron storage. [16] However, increased serum ferritin is not specific for increased body iron storage; systemic inflammation and renal failure can also increase serum ferritin levels. One explanation for the elevated levels of serum ferritin in our study is that ALS patients are exposed to chronic inflammation due to bronchial congestion. However, bronchial congestion is present only in patients with respiratory muscle weakness, most often at the end stages of the disease, and our patients underwent blood samples mainly early in the disease. Apart from bronchial congestion, inflammatory markers have been shown to be increased in ALS, but none were available for analysis in our study. [17] However, in a previous study, serum ferritin was increased in ALS patients in the absence of high CRP (C-Reactive Protein) levels, suggesting that inflammation may not be the only explanation for high ferritin in ALS. [10] In addition, high serum ferritin in our study was associated with increased TSC, a marker of iron overload. TSC is more likely to be reduced in circumstances of systemic inflammation. Intra cellular ferritin can be upregulated in response to oxidative stress, and serum ferritin could be increased in ALS patients due to oxidative stress. The poor prognosis of ALS patients with high level ferritin could reflect increased oxidative stress in these patients. However no data yet exist showing a relationship between serum ferritin (unlike intra cellular ferritin) and oxidative stress. Further, as for the inflammatory hypothesis, oxidative stress cannot explain the increased TSC in our patients.

Taken together, these observations suggest that ALS patients have increased serum ferritin levels due to increased body iron storage. A more specific test than serum ferritin for iron storage is needed to confirm our findings. The gold standard test, bone marrow biopsy, is too invasive for ALS patients, but hepatic MRI is one alternative. [18].

The pathophysiological mechanism that could explain why ALS patients have increased iron body storage, and how that is responsible for a decreased survival is, as yet, unknown. Genetic misregulation of iron intake could have a deleterious effect on motor neuron physiology and, associated with others factors, could initiate or worsen progression in ALS. The H63D mutation in the HFE gene was shown to be a risk factor for ALS in four independent studies, although no increased iron body storage is associated with this particular mutation. Even if the effect of H63D mutations on iron physiology is not clear, those studies indicate at least that a genetic misregulation of iron metabolism is a risk factor for ALS. On the other hand, elevated body iron storage in our ALS patients could reflect an unknown pathophysiological process in ALS. ALS can be seen as a multi-systemic with motor neuron degeneration being just one component of a widespread disease. [19] For example, in SOD1 mutated mice, energy metabolism is impaired, and levels of several hormones are modified. [20] Increased body iron storage could be part of this systemic spectrum, and could reflect the severity of ALS. Longitudinal studies of serum ferritin levels in ALS could help assess this hypothesis.

This study is, to our knowledge, the first to show that serum ferritin levels impacts survival in ALS. If elevated ferritin is related to increased body iron storage in ALS, the impact on survival could indicate that disruption of iron metabolism is involved in the pathophysiology of the disease. Further elucidation of the role of iron metabolism is needed, and drugs targeting iron metabolism, including iron chelation therapy, could by tried in clinical trials for ALS.

## Supporting Information

Table S1 IM variables levels in men according to disease status and age sub groups (<45 years old, between 45 and 60 years old, >60 years old) (means with standard deviations).
(DOC)

Table S2 IM variables levels in women according to disease status and age sub groups (<45 years old, between 45 and 60 years old, >60 years old) (means with standard deviations).
(DOC)

Table S3 IM variables levels according to disease status and gender (means with standard deviations).
(DOC)

Table S4 Minimum and maximum values of IM variables for each tertile group analysed in tables 4 and 5, with number of values.
(DOC)
