# Supplementary material for: Elevated Serum Ferritin Is Associated with Reduced Survival in Amyotrophic Lateral Sclerosis
Source: PLoS One. 2012 Sep 14;7(9):e45034. doi: 10.1371/journal.pone.0045034 (PMC3443244; doi:10.1371/journal.pone.0045034)
Supplement: Table S1 — IM variables levels in men according to disease status and age sub groups (<45 years old, between 45 and 60 years old, >60 years old) (means with standard deviations). (DOC) [file pone.0045034.s001.doc]

|  | Men | | | | | | | |
| --- | --- | --- | --- | --- | --- | --- | --- | --- |
|  | ALS | | | | Controls | | | |
|  | <45 | 45-60 | >60 | p value | <45 | 45-60 | >60 | p value |
| number of values (for serum iron) | 77 | 136 | 147 |  | 90 | 71 | 12 |  |
| serum iron (micromol/L) | 21.55 (6.99) | 19.76 (6.56) | 19.59 (6.35) | 0.08 | 18.89 (5.18) | 19.88 (4.99) | 20.55 (6.27) | 0.36 |
| serum transferrin (g/L) | 2.28 (0.37) | 2.27 (0.43) | 2.19 (0.40) | 0.17 | 2.37 (0.32) | 2.33 (0.31) | 2.39 (0.38) | 0.4 |
| saturation coefficient of transferrin (%) | 38.2 (14.65) | 35.24 (13.63) | 36.09 (13.77) | 0.33 | 32.57 (10.43) | 34.61 (9.61) | 34.87 (11.01) | 0.77 |
| serum ferritin (microg/L) | 202.53 (134.7) | 236.65 (153.6) | 235.77 (148.6) | 0.23 | 175.42 (73) | 186.04 (82.64) | 188.08 (95.36) | 0.66 |

Table S1. Means with standard deviations () of IM variables in men according to disease status and age sub groups (<45 years old, between 45 and 60 years old, >60 years old).
