# Supplementary material for: Elevated Serum Ferritin Is Associated with Reduced Survival in Amyotrophic Lateral Sclerosis
Source: PLoS One. 2012 Sep 14;7(9):e45034. doi: 10.1371/journal.pone.0045034 (PMC3443244; doi:10.1371/journal.pone.0045034)
Supplement: Table S2 — IM variables levels in women according to disease status and age sub groups (<45 years old, between 45 and 60 years old, >60 years old) (means with standard deviations). (DOC) [file pone.0045034.s002.doc]

|  | Women | | | | | | | |
| --- | --- | --- | --- | --- | --- | --- | --- | --- |
|  | ALS | | | | Controls | | | |
|  | <45 | 45-60 | >60 | p value | <45 | 45-60 | >60 | p value |
| number of values | 41 | 123 | 170 |  | 61 | 39 | 22 |  |
| serum iron (micromol/L) | 18.90 (7.98) | 18.92 (5.16) | 17.48 (5.4) | 0.07 | 18.61 (7.66) | 17.24 (6.01) | 17.20 (4.8) | 0.52 |
| serum transferrin (g/L) | 2.50 (0.56) | 2.39 (0.46) | 2.32 (0.45) | 0.66 | 2.60 (0.51) | 2.47 (0.4) | 2.44 (0.44) | 0.7 |
| saturation coefficient of transferrin (%) | 32.26 (17.23) | 32.50 (10.57) | 31.15 (13.03) | 0.07 | 30.22 (13.97) | 28.21 (9.66) | 28.87 (9.39) | 0.28 |
| serum ferritin (microg/L) | 56.04 (62.9) | 113.25 (97.71) | 135.91 (109) | <0.001* | 53.68 (55.8) | 92.07 (65.27) | 85.45 (39.68) | 0.003* |

Table S2. . Means with standard deviations () of IM variables in women according to disease status and age sub groups (<45 years old, between 45 and 60 years old, >60 years old)
