# Supplementary material for: Elevated Serum Ferritin Is Associated with Reduced Survival in Amyotrophic Lateral Sclerosis
Source: PLoS One. 2012 Sep 14;7(9):e45034. doi: 10.1371/journal.pone.0045034 (PMC3443244; doi:10.1371/journal.pone.0045034)
Supplement: Table S3 — IM variables levels according to disease status and gender (means with standard deviations). (DOC) [file pone.0045034.s003.doc]

|  | ALS | | | Controls | | |
| --- | --- | --- | --- | --- | --- | --- |
|  | Men | Women | p value | Men | Women | p value |
| number of values | 360 | 334 |  | 173 | 124 |  |
| serum iron (micromol/L) | 20.07 (6.59) | 18.18 (5.72) | <0.001* | 19.42 (5.18) | 17.98 (6.67) | 0.038* |
| serum transferrin (g/L) | 2.24 (0.41) | 2.37 (0.47) | <0.001* | 2.36 (0.32) | 2.53 (0.47) | <0.001* |
| saturation coefficient of transferrin (%) | 36.23 (13.92) | 31.78 (12.80) | <0.001* | 33.56 (10.13) | 29.43 (11.88) | 0.001* |
| serum ferritin (microg/L) | 228.56 (227.71) | 117.01 (103.00) | <0.001* | 180.65 (78.86) | 73.53 (62.06) | <0.001* |

Table S3. Means with standard deviations () of IM variables according to disease status and gender.
