# Supplementary material for: Elevated Serum Ferritin Is Associated with Reduced Survival in Amyotrophic Lateral Sclerosis
Source: PLoS One. 2012 Sep 14;7(9):e45034. doi: 10.1371/journal.pone.0045034 (PMC3443244; doi:10.1371/journal.pone.0045034)
Supplement: Table S4 — Minimum and maximum values of IM variables for each tertile group analysed in tables 4 and 5, with number of values. (DOC) [file pone.0045034.s004.doc]

|  | men | | | women | | |
| --- | --- | --- | --- | --- | --- | --- |
|  | low | medium | high | low | medium | high |
| serum iron (micromol/L) | 2-17 | 18-22 | 23-51 | 4-15 | 16-19 | 20-46 |
| number of values | 115 | 108 | 114 | 113 | 98 | 111 |
| serum transferrin (g/L) | 0.8-2 | 2.1-2.3 | 2.4-4.3 | 1.10-2.10 | 2.20-2.50 | 2.6-4.5 |
| number of values | 111 | 106 | 112 | 112 | 106 | 96 |
| transferrin saturation coefficient (TSC) (%) | 0.3-30 | 31-40 | 42-98 | 0.2-26 | 27-34 | 35-89 |
| number of values | 114 | 110 | 106 | 109 | 99 | 105 |
| serum ferritin (microg/L) | 15.77-132.8 | 136.12-263.11 | 264.77-889.76 | 7.47-63.91 | 64.74-123.67 | 125.33-587.64 |
| number of values | 99 | 101 | 99 | 98 | 99 | 98 |

Table S4. Minimum and maximum values of IM variables for each tertile group analysed in tables 4 and 5, with number of values.
